# Associations of sociodemographic and clinical factors with perinatal depression among Israeli women: a cross-sectional study

**DOI:** 10.1186/s12888-019-2311-4

**Published:** 2019-11-01

**Authors:** Limor Adler, Judith Tsamir, Rachel Katz, Gideon Koren, Ilan Yehoshua

**Affiliations:** 1 Family Medicine - Southern region, Maccabi Health Services, Tel Aviv University, Tel Aviv, Israel; 2 Maccabi Healthcare Services, Tel Aviv, Israel; 3 Kahn - Maccabi Institute of Research and Innovation, Ariel University, Ari’el, Israel; 4 Ben Gurion University, Be’er Sheva, Israel

**Keywords:** Perinatal depression, Edinburgh postnatal depression scale, Pregnancy, Postpartum, Sociodemographics status

## Abstract

**Background:**

Perinatal depression is a common problem that affects about 18% of women worldwide, though the heterogeneity between countries is great. The aims of this study were to assess the prevalence of perinatal depressive symptoms in a national sample of women in Israel, and to investigate associations of these symptoms with demographic, medical and lifestyle factors.

**Methods:**

The study included all members of Maccabi Health Services, the second largest health maintenance organization in Israel, who filled the Edinburgh Postnatal Depression Scale (EPDS) during 2015–2016. Crude odds ratios (ORs) and adjusted ORs (aORs) are presented for associations of sociodemographic, medical and lifestyle factors with perinatal depressive symptoms, according to a score ≥ 10 on the EPDS.

**Results:**

Of 27,520 women who filled the EPDS, 1346 (4.9%) met the criteria for perinatal depression. In a logistic regression analysis we found the following factors associated with perinatal depression: the use of antidepressant medications (aOR = 2.34, 95% CI 1.94–2.82, *P* < 0.001 and aOR = 3.44; 95% CI 2.99–3.97, P < 0.001 for ≤3 months and > 3 months respectively), a diagnosis of chronic diabetes mellitus (aOR = 2.04; 95% CI 1.22–3.43, *P* = 0.007), Arab background (aOR = 2.28; 95% CI 1.82–2.86, *P* < 0.001), current and past smoking (aOR = 1.62; 95% CI 1.35–1.94, P < 0.001 and aOR = 1.36; 95% CI 1.05–1.76, *P* = 0.021, respectively), and anaemia (aOR = 1.17; 95% CI 1.04–1.32, *P* = 0.011). Orthodox Jewish affiliation and residence in the periphery of the country were associated with lower perinatal depression (aOR = 0.48; 95% CI 0.36–0.63, *P* < 0.001 and aOR = 0.72; 95% CI 0.57–0.92, *P* = 0.007, respectively).

**Conclusions:**

The prevalence of perinatal depression in this study was 4.9%. Perinatal depression was associated with a number of demographic, medical and lifestyle factors, including the use of antidepressant medication, chronic diabetes mellitus, Arab background, current or past smoking, and anaemia. These risk factors may help identify women at risk of perinatal depression.

## Background

Women’s physical and mental health during the perinatal period may impact their functioning, quality of life, parenting capability and subsequent pregnancies, as well as the health and well-being of their children [1]. Postpartum depression was stated as a specifier of depression in the Diagnostic and Statistical Manual of Mental Disorders 4th edition (DSM-IV) [2]. In the DSM-V [3], the specifier was defined as perinatal depression, and thus includes depression during pregnancy; however, the 4-week postpartum cutoff remained.

A recently published systematic review and meta-analysis of 291 studies that used the Edinburgh Postnatal Depression Scale (EPDS) reported global pooled prevalence of perinatal depression of 17.7% (95% confidence interval: 16.6–18.8%) [4]. Heterogeneity was substantial across the 56 countries included (Q = 16,823, *p* = 0.000, I2 = 98%); depression ranged from 3% (2–5%) in Singapore to 38% (35–41%) in Chile. The rates for the Israeli studies included in the review ranged from 5.2 to 43.0%.

Several risk factors have been found to be associated with perinatal depression, including socio-demographic (maternal age, low socioeconomic status), social (stressful life events, social support), medical (a previous history of depression, low hemoglobin level, higher blood glucose level), obstetrics (history of miscarriages, parity) and lifestyle factors (smoking) [5].

Perinatal depression has been reported to be associated with increased risk of recurrence of depression [6], later psychiatric morbidity and suicide [7, 8], as well as with negative effects on offspring. A meta-analysis of 193 studies reported associations of maternal depression with poorer behavior and emotional functioning among children born from such pregnancies [9]. More recent publications have documented diverse adverse infant and child outcomes of perinatal depression in both high income [10] and low and middle-income countries [11]. Identifying risk factors of perinatal depression may potentially help healthcare professionals better identify women with depression, by increasing vigilance regarding women who have these risk factors.

According to the protocol of the Israel Ministry of Health, all pregnant women should fill the EPDS during pregnancy (after gestational week 26) and again after delivery (4–9 weeks postpartum).

The healthcare system in Israel is public and comprises four healthcare funds. Each resident chooses a healthcare fund; the payments are equal. The characteristics of the populations in all the funds are similar, and thus the population in our study is likely a good representative of the general population in Israel.

Many of the studies conducted in Israel on perinatal depression specifically examined the Arab population or religious Jewish women [12–14]. The studies that were done on the general population were conducted more than one decade ago [15–18], and did not compare differences between population sub-groups.

The objectives of this study were to assess the prevalence of perinatal depressive symptoms in a national sample of women from one healthcare fund, and to investigate associations of demographic, medical and lifestyle factors with perinatal depressive symptoms.

## Methods

### Design

This cross-sectional study is based on mining of the electronic patient database of the second largest health fund in Israel, Maccabi Health Services (MHS). Data of sociodemographic, medical and lifestyle factors were collected for the women included, for the period of the pregnancy and 6 weeks postpartum.

### Setting and population

Nursing staff are responsible for administering the EPDS in mother and child clinics in Israel; 48% of these clinics are governmental, 48% belong to the healthcare funds and 4% are municipal. All women in MHS who filled the EPDS in the healthcare fund clinics during 2015–2016 were included in the current study. Data from governmental and municipal mother and child clinics were not available for this study.

### Variables

The outcome variable of perinatal depression was based on the EPDS. The EPDS [19] is a validated, easily administered and widely used scale that was designed specifically to assess perinatal depression. This scale was recommended for assessment of perinatal depression by a systematic review [20]. Among its strengths are its brevity and the absence of the word “depression”.

The EPDS comprises 10 questions that access information about the respondent’s mood and depressive symptoms during the 7 days preceding its administration. The response to each question is scored 0–3; thus, the highest possible score is 30. In this study, a score of ≥10 was classified as depression. We used one EPDS score for every woman who filled the EPDS (if filled twice, the lower score was taken to ensure a conservative estimate).

Although the DSM-V states a cutoff point of 4 weeks for perinatal depression, we included in this study all women who filled the EPDS during pregnancy and until 9 weeks postpartum, according to the protocol of the Israel Ministry of Health.

Predictor variables accessed included sociodemographic, medical and lifestyle factors.

The sociodemographic factors examined were age [< 25, 25–40, > 40 years], living in the peripheral region of the country (yes/no), socioeconomic status (SES) (categorical) and population group [Arab, Orthodox Jew, other]). The classification of residence in a peripheral area is determined by geographical distance from the center of the country and distance from large cities (based on a measure of the Israel Central Bureau of Statistics). SES was determined according to residence. In 2008, the state of Israel was divided into 210 localities, for the calculation of SES. Each locality was ranked from 1 to 10 (10 represents the highest SES), according to 16 factors, including education, employment, and car ownership.

The medical factors examined were medical conditions (cardiovascular disease [yes/no], chronic diabetes mellitus [yes/no], hypertension [yes/no]), medications (the use of antidepressant medications [none, ≤3 months, > 3 months]), and blood test results (haemoglobin [≤10.5 d/dl or > 10.5 g/dl], ferritin, iron, vitamin B12, folic acid, vitamin D, TSH and c-reactive protein [continuous variables]).

Medical conditions and blood tests were considered according to information recorded in the files of the women included in the study. Iron, ferritin, vitamin D, vitamin B-12, folic acid, C-reactive protein and thyroid stimulating hormone (TSH) were not tested in all pregnant women; therefore, data were available for only some of them. If more than one value was available for a given parameter, the lowest value was considered in the analysis, except for TSH, for which both minimal and maximal values for each woman were entered into the analysis. In this study there was no access to measures of glucose and the glucose tolerance test.

The lifestyle factors examined were: smoking habits (never, past smoking, current smoking), as recorded by medical staff.

### Statistical analysis

A descriptive analysis of the participants was conducted. Characteristics of the women who filled the EPDS were compared with all members of MHS who delivered in the study period. This was to assess the similarity of the sample population with the general population of parturients, in regard to age, residence in the periphery and population group (Arabs, orthodox Jewish).

Univariate analysis was performed to compare women who were classified as having perinatal depressive symptoms (a score ≥ 10 on the EPDS test) and those without these symptoms. The Chi square test was used to investigate associations of categorical variables. The Independent T test was used for comparing normally distributed data. A *p*-value of less than 0.05 was considered statistically significant. Crude odds ratios (ORs) were based on the univariate analysis. Variables that were statistically significant in the univariate analysis were entered into a logistic regression analysis that assessed adjusted odds ratios (aORs) for associations with perinatal depression symptoms according to the EPDS. The Statistical Package for Social Sciences (SPSS) software version 21 was used for data analysis.

## Results

### Descriptive analysis

During 2015–2016, 81,693 babies were born to women who were members of MHS; about half of these women went to mother and child clinics in MHS, according to reports of these clinics. Of them, 27,520 filled the EPDS (estimated 70%). Women who did and did not fill the EPDS were similar in age and similar proportions lived in the periphery. The proportions of Arabs and Orthodox Jews who filled the EPDS were smaller than their proportions among all women who delivered during the study period (Table 1).

**Table 1:** Women who filled the Edinburgh Postpartum Depression Scale (EPDS) compared to all members of Maccabi Health Services (MHS) who gave birth during 2015–2016

Characteristics	Members of MHS who filled the EPDS in MHS during 2015–2016 *n* = 27,520	All members of MHS who gave birth during 2015–2016 *n* = 81,693	*P*-value
Age, years (mean ± SD)	33.2 (±5.63)	32.4 (±6.17)	< 0.001
Periphery	8.0%	7.5%	0.007
Arabs	4.1%	6.3%	< 0.001
Orthodox Jews	9.9%	16.2%	< 0.001

Among the 27,520 women who filled the EPDS, for 21,448 (77.9%), the point of time during the perinatal period that the questionnaire was filled was known. Of these, 8240 (29.9%) filled the questionnaire during pregnancy and 13,208 (48%) filled the questionnaire after delivery. For those who filled the questionnaire after delivery, the median number of days after delivery was 35 (interquartile range: 32–43).

Of the 27,520 women who filled the EPDS, 1346 (4.9%) were classified as having perinatal depression. The mean age of women with and without depression was similar.

### Univariate analysis

Several factors were found to be independently associated with perinatal depression in the univariate analysis (Table 2). Regarding sociodemographic factors, age > 40 years and Arab background were positively associated with perinatal depressive symptoms; residence in the periphery of the country and Orthodox Jewry were negatively associated with these symptoms. Regarding medical factors, chronic diabetes mellitus, a history of antidepressant medication use, anaemia, low ferritin and low iron were positively associated with perinatal depressive symptoms. Regarding lifestyle factors, current and past smoking were associated with perinatal depression symptoms.

**Table 2:** A univariate analysis of demographic and clinical characteristics of women who did and did not have perinatal depression according to the Edinburgh Postpartum Depression Scale

Characteristics	with perinatal depression *n* = 1346 (4.9%)		without perinatal depression *n* = 26,174 (95.1%)		Crude OR (95% CI)	*P*-value
	*N*	Percentage	*N*	Percentage		
Sociodemographic factors (data provided for all women)						
Age (years)						
Mean ± SD	33.2 (±5.6)		33.5 (±5.8)		1.01 (1.001–1.02)	0.026
< 25	84	6.3	1675	6.4	1.02 (0.81–1.29)	0.834
25–40	1082	80.4	21,672	82.8	Reference	
> 40	180	13.3	2827	10.8	1.27 (1.07–1.50)	0.005
Periphery	79	6.1	2041	8.0	0.74 (0.59–0.93)	0.011
Population group						
Arabs	108	8.1	994	3.8	2.1 (1.70–2.58)	< 0.001
Orthodox Jews	61	4.5	2587	9.9	0.45 (0.35–0.59)	< 0.001
Others	1177	87.4	22,593	86.3	Reference	
Socioeconomic status (median, range)	6.0 (0–10)		6.0 (0–10)		1.01 (0.98–1.04)	0.760
Medical conditions (data provided for all women)						
Cardiovascular disease	25	1.9	364	1.4	1.35 (0.89–2.03)	0.151
Chronic Diabetes Mellitus	18	1.4	149	0.6	2.38 (1.45–3.89)	0.001
Hypertension	27	2.1	401	1.6	1.32 (0.89–1.96)	0.163
Medications (data provided for all women)						
Antidepressant use						
None	868	64.5	22,167	84.7	Reference	
≤ 3 months	153	11.4	1639	6.3	2.38 (1.99–2.85)	< 0.001
> 3 months	325	24.1	2368	9.0	3.50 (3.06–4.01)	< 0.001
Blood Tests, N-number of women tested						
Haemoglobin – N	1296	96.3	25,394	97.0		0.124
Mean ± SD (g/dl)	10.91 (±0.94)		10.96 (±0.89)		0.94 (0.88–0.99)	0.047
≤ 10.5 (Anaemia)	427	32.9	7570	29.8	1.16 (1.03–1.30)	0.016
> 10.5	869	67.1	17,824	70.2		
Ferritin – N	859	63.8	15,601	59.6		0.002
Mean ± SD (ng/ml)	33.84 (±31.77)		37.69 (±44.77)		0.997 (0.995-0.999)	.001
Iron – N	676	50.2	11,397	43.5		< 0.001
Mean ± SD (mcg/dl)	67.88 (±35.53)		71.87 (±37.02)		0.998 (0.996–1)	0.041
B12 - N	561	41.7	9378	35.8		< 0.001
Mean ± SD (pg/ml)	361.18 (±157.53)		361.16 (±148.69)		1 (0.99–1.0)	0.997
Folic acid – N	627	46.6	10,294	39.3		< 0.001
Mean ± SD (ng/ml)	2.30 (±3.29)		2.35 (±3.33)		0.99 (0.97–1.02)	0.700
Vitamin D (min) – N	755	56.1	13,853	52.9		0.023
Mean ± SD (ng/ml)	20.40 (±9.09)		20.44 (±9.15)		1 (0.99–1.01)	0.915
TSH (min) - N	1260	93.6	23,915	91.4		0.004
Mean ± SD (mlU/l)	1.33 (±0.87)		1.35 (±0.83)		0.97 (0.91–1.04)	0.427
TSH (max) - N	1260	93.6	23,915	91.4		0.004
Mean ± SD (mlU/l)	3.12 (±8.18)		2.82 (±5.82)		1.007 (0.99–1.01)	0.198
C-reactive protein - N	332	24.7	5361	20.5		< 0.001
Mean ± SD (mg/dl)	0.99 (±2.35)		1.00 (±2.41)		0.997 (0.95–1.04)	0.909
Lifestyle factors (data provided for all women)						
Smoking						
Never	1111	82.5	23,301	89.0	Reference	
Current	163	12.1	1843	7.0	1.85 (1.56–2.20)	< 0.001
Past	72	5.3	1030	3.9	1.47 (1.15–1.88)	0.002

Factors that were not found to be associated with perinatal depression in the univariate analysis included SES and several medical factors that were examined: cardiovascular disease, chronic hypertension and lower levels of vitamin B12, folic acid, vitamin D, c-reactive protein and TSH.

### Multivariate regression analysis

A logistic regression analysis included 27,520 women (Table 3). Most blood tests were not included in the logistic regression due to the high level of missing data (about 50%).

**Table 3:** Logistic regression of perinatal depression symptoms according to sociodemographic, medical and lifestyle characteristics

Variable	Adjusted OR (95% CI)	*P* value
Sociodemographic factors		
Age		
Age 25–40 years	Reference	
Age < 25 years	1.26 (0.99–1.61)	0.065
Age > 40 years	1.09 (0.92–1.30)	0.316
Residence in the periphery of the country	0.72 (0.57–0.92)	0.007
Population group		
Non Arab, non Orthodox-Jewish population	Reference	
Arab background	2.28 (1.82–2.86)	< 0.001
Orthodox-Jewish affiliation	0.48 (0.36–0.63)	< 0.001
Medical conditions		
Chronic Diabetes Mellitus	2.04 (1.22–3.43)	0.007
Medications		
No prior use of antidepressant medication	Reference	
Antidepressant medication < 3 m	2.34 (1.94–2.82)	< 0.001
Antidepressant medication > 3 m	3.44 (2.99–3.97)	< 0.001
Blood Tests		
Anaemia (Haemoglobin > 10.5)	1.17 (1.04–1.32)	0.011
Lifestyle factors		
Never smoked	Reference	
Current smoking	1.62 (1.35–1.94)	< 0.001
Past smoking	1.36 (1.05–1.76)	0.021

Three sociodemographic factors were significant in the multivariate analysis. Women living in the periphery of the country (aOR = 0.72; 95% CI 0.57–0.92, *P* = 0.007) and Orthodox Jewish women (aOR = 0.48; 95% CI 0.36–0.63, *P* < 0.001) were less likely to report perinatal depressive symptoms. Women from Arab background were more likely to report perinatal depressive symptoms (aOR = 2.28; 95% CI 1.82–2.86, *P* < 0.001). Age was not associated with perinatal depressive symptoms in the multivariate analysis.

Regarding medical factors, women with chronic diabetes mellitus (aOR = 2.04; 95% CI 1.22–3.43, *P* = 0.007), anaemia (aOR = 1.17; 95% CI 1.04–1.32, *P* = 0.011) and a history of antidepressant medication use (aOR = 2.34, 95% CI 1.94–2.82, *P* < 0.001 for treatment of 3 months or less; and aOR = 3.44; 95% CI 2.99–3.97, *P* < 0.001 for treatment of more than 3 months) were more likely to report perinatal depressive symptoms.

Regarding lifestyle factors, women who were current or past smokers compared to never smokers were more likely to report perinatal depressive symptoms (aOR = 1.62; 95% CI 1.35–1.94, P < 0.001 and aOR = 1.36; 95% CI 1.05–1.76, *P* = 0.021, respectively).

## Discussion

**Fig. 1:**
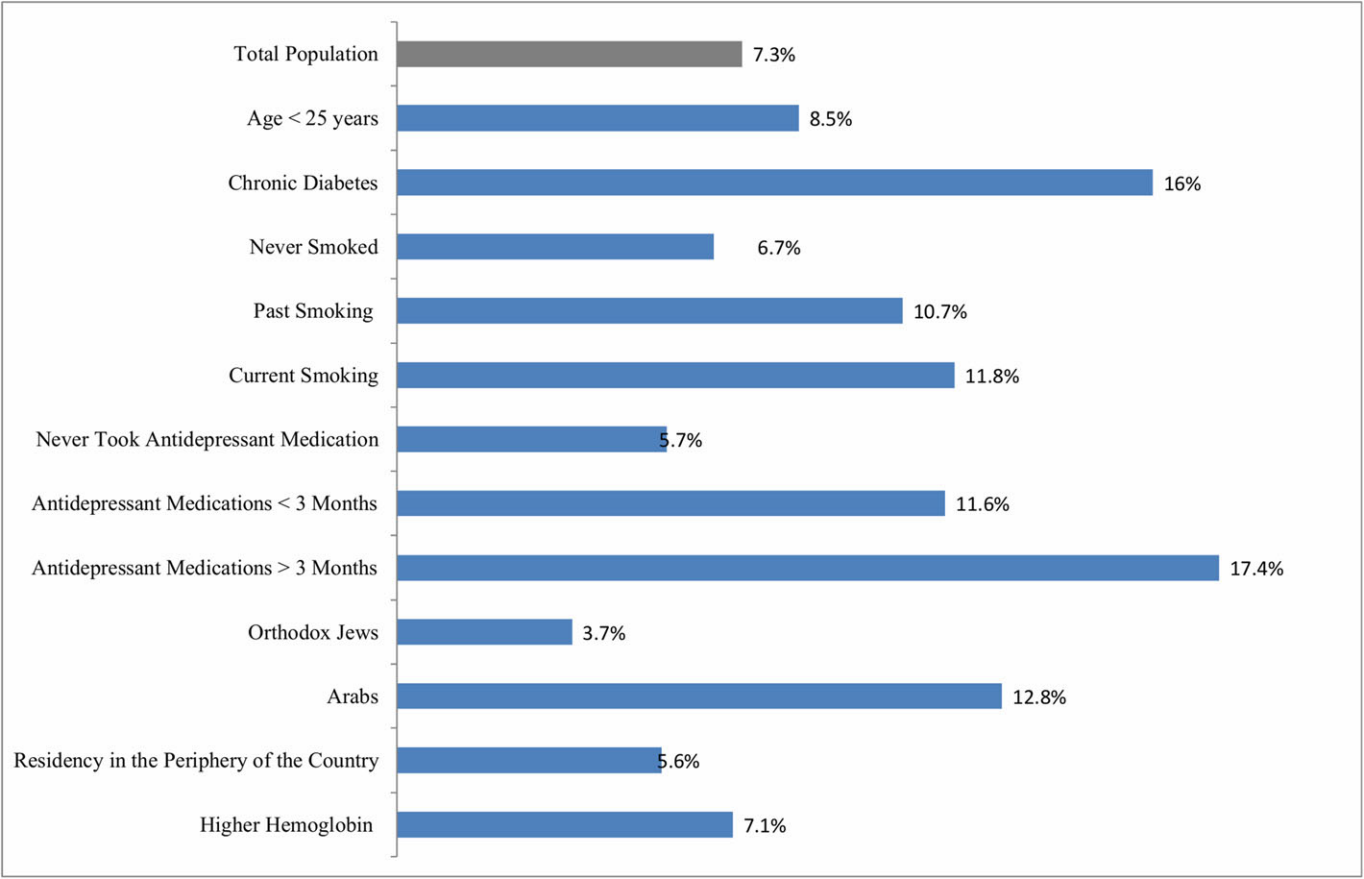
Incidence of symptoms of perinatal depression by sub-population

In this population-based study, 4.9% of women who filled the EPDS during pregnancy or after delivery reported perinatal depression symptoms (EPDS score ≥ 10). Prior treatment with antidepressant medication, particularly for a period greater than 3 months, Arab background, current or past smoking, a diagnosis of diabetes mellitus, and anaemia (haemoglobin ≤10.5 d/dl) were associated with increased odds of having perinatal depression. Orthodox Jewish affiliation and residence in the periphery of the country appeared as protective factors for perinatal depressive symptoms (Fig. 1).

The 4.9% rate of perinatal depression symptoms reported here was lower than rates reported in other studies in Israel [4]. This discrepancy may be due to differences in study design. Our study was nationwide and based on data collected from the community. Women with depressive symptoms may be more reluctant to attend mother and child clinics. One of the difficulties in comparing between studies, particularly studies conducted in different countries, is that cutoff points of the EPDS may vary, in the range of 9–14. For the current study, we set 10 as the cutoff, as is determined by the Israel Ministry of Health. This cut-off was used in the majority of studies [4], and was recommended in the development of the EPDS [19].

Prior use of antidepressant medications was the factor most strongly associated with perinatal depressive symptoms in this study. This concurs with an association of prior depression with postpartum depression reported by studies conducted over one and a half decades ago [21], as well as with studies conducted in 42 countries during 2005–2014 [22].

A diagnosis of chronic diabetes mellitus increased the odds of perinatal depressive symptoms in the present study, by more than two-fold. EPDS scores ≥10 were found in 11.0% of the women with diabetes, compared to 4.9% of all the women who completed the questionnaire. Numerous studies have described an association between diabetes and depression [23], and particularly during pregnancy [24]. This relation can be understood in light of the acute stress of maintaining balanced glucose control during pregnancy. We note that the definition of diabetes in the current study referred to chronic disease (type 1 or type 2) and that we did not have any data about gestational diabetes.

The risk of perinatal depression was greater for Arab women and less for Orthodox women, compared to the remaining population. This finding concurs with the inverse association of orthodox affiliation with postpartum depressive symptoms, as assessed by the EPDS, in a sample of 327 Israeli women [12]. Understanding characteristics of Orthodox communities may help explain their lower scores on the EPDS. For one, the view that pregnancy and childbirth are blessed and that fertility is central to a woman’s identity may help reduce the risk of depression. In addition, the high level of community support may alleviate some of the burden following childbirth. On the other hand, Orthodox women may be relunctant to acknowledge feeling depressed due to the negative effect that depression could have on matchmaking possibilities for future generations. Thus, seeking help for postpartum depression may be even lower among Orthodox women than the low rate that has been reported for women in general [25, 26]. Finally, Orthodox women generally stay close to their family of origin, contrasting with Arab women who generally join their husband’s family. Arab women share some characterstics with Orthodox Jewish women (a religious minority, a high birth rate and social stigma regarding depression). However, a number of characteristics that are unique to Arab women may help elucidate the discrepancy in results between the groups. The Arab population has a mean lower income, lower socioeconomic status, lower accessibility to health services, and higher rates of infant mortality and congenital disorders [27]. Similar results to those presented here were seen in a study done on Bedouin women (sub group of Arab population) that showed substantially higher rates of perinatal depression compared to the general population in Israel [13].

In the current cohort, anaemia (haemoglobin ≤10.5 g/dl) was associated with perinatal depressive symptoms. Our findings concur with cohort studies conducted in Sweden [28] and Iran [29] that showed associations of anaemia at delivery and at discharge from the maternity ward, respectively (haemoglobin < 110 g/L) with postpartum depressive symptoms (EPDS≥12 and EPDS > 13, respectively). However, among Chinese women, no relationship was found between depressive symptoms according to the EPDS and either anaemia or iron [30].

Despite the protocol of the Israel Ministry of Health, which states that all pregnant women should fill the EPDS during pregnancy and again after delivery, in this study we estimated that 30% of women did not fill the EPDS at all. Perinatal depression has many potential effects on the physical and mental health of mothers and children, and thus it should be a main focus of community healthcare services. Increasing the proportion of women who fill the EPDS is an important first step in identifying women at risk.

The current study contributes to the understanding of the phenomenon of perinatal depression in Israel, a country with a relative high birth rate. The findings may help physicians better identify women at increased risk for perinatal depression. The strengths of this study are the large cohort and the availability of important data, such as prior use of antidepressant medication and haemoglobin level for all the women included. Limitations include selection bias and missing data for a number of the variables investigated, including the timing of filling the questionnaire (missing for 23% of women), the presence of gestational diabetes and the number of previous children. Future research is needed to assess if the recognition of risk factors will promote early diagnosis of women with perinatal depression, and whether this will lead to improved treatment for women with these symptoms.

## Conclusions

In this study, 4.9% of women who filled the EPDS during pregnancy or after delivery reported perinatal depressive symptoms. Several factors appear to increase the risk of perinatal depression including sociodemographic factors (Arab background), medical factors (prior use of antidepressant medication, chronic diabetes mellitus and anaemia) and lifestyle factors (smoking). Several factors appear to decrease the risk of perinatal depression, including sociodemographic factors (residence in the periperhy of the country and Orthodox-jewish affiliation).

## Data Availability

The datasets used and analyzed during the current study are available from the corresponding author on reasonable request.

## Abbreviations

aORs: Adjusted odds ratios
DSM: Diagnostic and Statistical Manual of Mental Disorders
EPDS: Edinburgh Postnatal Depression Scale
MHS: Maccabi Health Services
ORs: Crude odds ratios
SES: Socioeconomic status
TSH: Thyroid stimulating hormone
